# *Salmonella* Risk Assessment in Poultry Meat from Farm to Consumer in Korea

**DOI:** 10.3390/foods12030649

**Published:** 2023-02-02

**Authors:** Hyemin Oh, Yohan Yoon, Jang-Won Yoon, Se-Wook Oh, Soomin Lee, Heeyoung Lee

**Affiliations:** 1 Risk Analysis Research Center, Sookmyung Women’s University, Seoul 04310, Republic of Korea; 2 Department of Food and Nutrition, Sookmyung Women’s University, Seoul 04310, Republic of Korea; 3 College of Veterinary Medicine & Institute of Veterinary Science, Kangwon National University, Chuncheon 24341, Republic of Korea; 4 Department of Food and Nutrition, Kookmin University, Seoul 02703, Republic of Korea; 5 Food Standard Research Center, Korea Food Research Institute, Wanju 55365, Republic of Korea

**Keywords:** poultry meat, *Salmonella*, risk assessment, farm to consumer, food safety

## Abstract

This study predicted *Salmonella* outbreak risk from eating cooked poultry in various methods. The incidence of *Salmonella* in poultry meat and the environment from farm to home for consumption was investigated. To develop the predictive models, *Salmonella* growth data were collected at 4–25 °C during storage and fitted with the Baranyi model. The effects of cooking on cell counts in poultry meat were investigated. Temperature, duration, and consumption patterns were all searched. A simulation in @Risk was run using these data to estimate the probability of foodborne *Salmonella* disease. In farm, *Salmonella* was detected from only fecal samples (8.5%; 56/660). In slaughterhouses, *Salmonella* was detected from feces 16.0% (38/237) for chicken and 19.5% (82/420) for duck) and from carcasses of each step (scalding, defeathering, and chilling) by cross contamination. In chicken (*n* = 270) and duck (*n* = 205), *Salmonella* was detected in 5 chicken (1.9%) and 16 duck meat samples (7.8%). *Salmonella* contamination levels were initially estimated to be −3.1 Log CFU/g and −2.5 Log CFU/g, respectively. With *R^2^* values between 0.862 and 0.924, the predictive models were suitable for describing the fate of *Salmonella* in poultry meat with of 0.862 and 0.924. The *Salmonella* was not detected when poultry meat cooks completely. However, if poultry meat contaminated with *Salmonella* were cooked incompletely, *Salmonella* remained on the food surface. The risk of foodborne *Salmonella* disease from poultry consumption after cooking was 3.0 × 10^−10^/person/day and 8.8 × 10^−11^/person/day in South Korea, indicating a low risk.

## 1. Introduction

*Salmonella* is a Gram-negative, rod-shaped bacterium that can survive without oxygen and causes diseases in humans. Most infections are due to the ingestion of foods contaminated by animal feces, or by environmental pollutions. *Salmonella* is the most common bacterial pathogen causing gastrointestinal infection worldwide [1,2]. In the European Union, the annual incidence rate of foodborne outbreaks caused by *Salmonella* was relatively high at 14.3% in 2021 as compared with 2020 [3]. There were approximately 5581 outbreaks of foodborne illness reported in Korea from 2002 to 2021, affecting 136,162 patients [4]. *Salmonella* infection was associated with 413 outbreaks involving 16,475 patients, making it the third most prevalent cause of foodborne outbreaks after norovirus and enteropathogenic *Escherichia coli* (EPEC) [4]. *Salmonella* is the second largest cause of death in the United States, resulting in 3 to 8 deaths annually [5]. 

*Salmonella* foodborne outbreaks occur in a variety of foods [6,7,8], according to CDC data, but they are most frequently found in poultry, including chicken, duck, and eggs. In addition, *Salmonella* is prevalent throughout the poultry supply chain, from slaughterhouse processing to retail and consumption [9,10,11]. Ren et al. (2016) [12] studied the microbiological quality of chicken and observed that farm, slaughterhouse, and retail market *Salmonella* prevalence was 7.0, 62.9, and 54.7%, respectively. Another study [13] reported that *Salmonella* was observed in high prevalence in retail meat shops (46.3%), commercial broiler farms (19.2%), and hatcheries (10.3%). These results indicate that *Salmonella* contaminates poultry meat at high level during the stage of production and retail chain. Therefore, it is crucial to evaluate the critical control points for chicken production and processing from the perspective of food safety. Additionally, there is substantial danger of *Salmonella* infection if it is not adequately heated before consumption. Raw or undercooked poultry or eggs cause 75% of *Salmonella* outbreaks, according to the CDC [14]. The risk of *Salmonella* contamination is low for hard-cooked meat, but medium and soft-cooked meat pose the possibility of salmonellosis [15,16]. 

Quantitative microbial risk assessment (QMRA) is performed to support scientific evidence for managing food safety [17,18], and it estimates the probability of foodborne pathogens in food during each stage connected to food preparation and consumption [19,20]. In this study, a QMRA scenario for *Salmonella* in chicken and duck was established, including the stages of manufacturing, home consumption, and cooking. QMRA used Korean circumstances and cooking procedures that are common in Korean chicken and duck recipes in this investigation. Therefore, the goal of this research was to evaluate the potential for *Salmonella*-related foodborne diseases from cooked poultry in Korea.

## 2. Materials and Methods 

### 2.1. Model Overview

**Figure 1:**
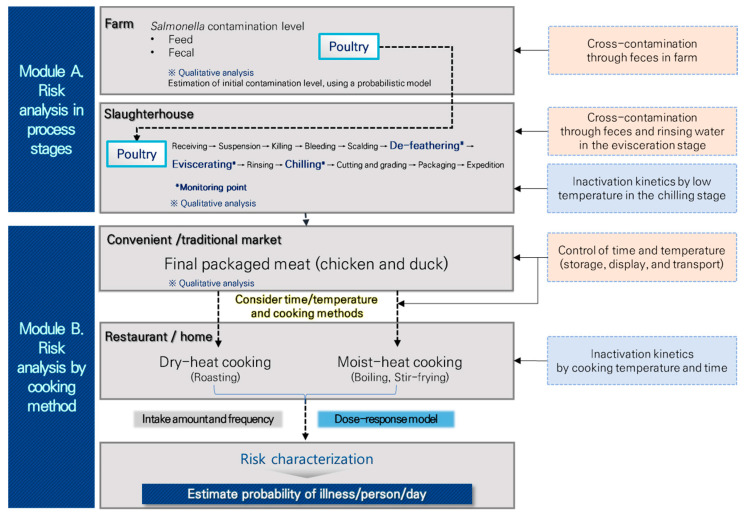
From farm to table, a flowchart illustrating the risk assessment model for *Salmonella* spp. in chicken and duck.

The prevalence and concentration of *Salmonella* in chicken and duck from farms to slaughterhouse and to home consumption were applied into the QMRA model. The model consisted of two modules; (A) risk factor analysis in the production stages and (B) risk analysis by cooking methods were both considered when evaluating the risk of *Salmonella* exposure (Figure 1). In module (A), the prevalence and contamination of *Salmonella* in farms and slaughterhouses, which are production stages, were investigated, and major risk factors were presented. In module (B), the risk of *Salmonella* in the process of transport, storage, and display of the products to supermarkets and traditional markets in the distribution stage, and consumption after cooking at home by consumers was determined by @Risk (Palisade Corp., Ithaca, NY, USA). Using 10,000 random repetitions, the possibility of *Salmonella*-related foodborne disease from consuming chicken and duck was estimated.

### 2.2. Salmonella Prevalence in Poultry

For *Salmonella* monitoring throughout the entire supply process of chickens and ducks, samples from the production stage were collected at poultry farms and slaughterhouses by region, and packaged chicken and duck meat at the distribution stage were purchased from supermarkets and traditional markets. Feed and fecal samples were collected from 18 poultry farms (two in the capital region, four in the Chungcheong region, five in the Gangwon region, three in the southwestern region, and four in the southeastern region). Furthermore, carcasses and fecal samples were collected by swab at 10 slaughterhouses (three in the capital region, one in the Chungcheong region, two in the Gangwon region, three in the southwestern region, and one in the southeastern region) for 6 stages (suspension, before/after defeathering and rinsing, evisceration, and chilling). At the farm, 610 and 660 feed and fecal samples were collected, respectively. In the suspension, before/after defeathering, and rinsing steps at slaughterhouses, 180 samples (160 samples from chicken slaughterhouses; 20 samples from duck slaughterhouses) were collected. In addition, 200 samples (180 samples from chicken slaughterhouses; 20 samples from duck slaughterhouses) were collected at each of the before/after chilling steps. A total of 657 fecal samples (237 samples from chickens; 420 samples from ducks) were collected from the evisceration step. A total of 270 raw chicken meats and 205 raw duck meats were purchased and investigated to detect *Salmonella* in 4 supermarkets and 20 traditional markets (six in the capital region, three in the Chungcheong region, three in the Gangwon region, four in the southeastern region, and four in the southwestern region). “Food Code [21]” and “Processing standards and ingredient specifications for livestock products [22]” were used to isolate and identify *Salmonella*. Twenty-five grams of each sample (meat, feed, and visceral samples) was uniformly collected and used as a sample. For sampling at the slaughterhouse, 0.5–1.0 g of feces or a cotton swab was dissolved in 1 mL of sterile phosphate-buffered saline (PBS, 8.0 g of NaCl, 1.5 g of Na_2_HPO_4_, 0.2 g of KCl, and 0.2 g of KH_2_PO_4_ in 1 L of distilled water; pH 7.4), vortexed, and 200 μL was used as a feces sample. The feces samples were added to 10 mL of buffered peptone water (BPW; Becton, Dickinson and Company, BD, Sparks, MD, USA). The 25 g of samples (meat, feed, and water) were deposited in sterile filter bags (3M, St. Paul, MN, USA) containing 225 mL BPW. The homogenates were then incubated for 18–24 h at 36 ± 1 °C. Then, 1 mL of this pre-enriched solution was added to 10 mL of Tetrathionate (TT; Becton, Dickinson and Company, BD, Sparks, MD, USA) broth and 10 mL of Rappaport Vassiliadis (RV; Becton, Dickinson and Company, BD, Sparks, MD, USA), and the mixtures were incubated at 36 ± 1 °C and 42 °C, respectively, for 20–24 h. The cultures were incubated at 37 °C for 20–24 h after being streaked onto xylose lysine deoxycholate agar (XLD agar; Becton, Dickinson and Company, Sparks, MD, USA) and Brilliant Green sulfa (BG sulfa; Becton, Dickinson and Company, Sparks, MD, USA) agar. *Salmonella* spp., indicated by black colonies with clear membranes on XLD agar and pink colonies on BG sulfa agar, were isolated and identified using VITEK’s biochemical test. *Salmonella* spp. strains were identified and cultured in tryptic soy broth (TSB; Becton, Dickinson and Company, Sparks, MD, USA) for 24 h at 37 °C. After bacterial growth, 150 μL of the culture was combined with 150 μL of 40% glycerol and stored at −80 °C.

### 2.3. Preparation of Salmonella Inoculum

Fourteen *Salmonella* strains isolated from chicken and duck and two reference strains (*S.* Typhimurium ATCC 700720 and *S.* Enteritidis ATCC 13076) were cultivated for 24 h in TSB. Then, 0.1 mL aliquots of cultures were transferred to 10 mL of TSB and incubated for another 24 h at 37 °C. The cell pellets were washed twice with PBS after 15 min of centrifugation at 1912× *g* and 4 °C. Optical density (OD) values of 600 nm cell suspensions were adjusted to achieve a *Salmonella* inoculum concentration of 6 Log CFU/mL.

### 2.4. Predictive Models Development

Predictive models were developed to describe the kinetic behavior of *Salmonella* by dipping 25 g of chicken samples in the *Salmonella* inoculum for 15 min and left for 5 min. The samples were stored at 4 °C, 10 °C, 15 °C, or 25 °C under aerobic conditions for up to 6 days (the same as a commercial product on the market). To count the *Salmonella* cells, 50 mL of 0.1% BPW was added to the samples and pummeled for 60 s at appropriate sampling intervals. Aliquots of 0.1 mL were taken from the homogenates and spread-plated onto XLD agar. After 24 h of inoculation at 37 °C, the colonies were counted. *Salmonella* cell counts were fitted using DMfit software (Institute of Food Research, Norwich, UK) and the Baranyi model [23]. The maximal specific growth rate (*μ_max_*; Log CFU/g/h) and lag-phase duration (LPD; h) were determined as kinetic parameters. The Baranyi model was as follows:

$$N_{t}=N_{0}+\mu_{max}\times ln\left[1+\frac{\exp\left(\mu_{max}\times A_{t}\right)-1}{\exp\left(N_{max}-N_{0}\right)}\right]$$

$$A_{t}=t+\frac{1}{\mu_{max}}ln\left(\frac{\exp\left(-\mu_{max}\right)+q_{0}}{1+q_{0}}\right)$$

$$q_{0}=\frac{1}{\exp\left(h_{0}\right)-1}$$

Using the polynomial model, the LN(LPD) and $\sqrt{\mu_{max}\;}$ values were examined as a function of temperature to develop a secondary model:

$$\mathrm{LN}(\mathrm{LPD})=a_{0}+a_{1}T^{1}+a_{2}T^{2}\;\mathrm{and}\;{\mu_{\max}}^{0.5}=a_{0}+a_{1}T^{1}$$

where *a_i_* and *T* represent the coefficients and storage temperature (°C), respectively. Additional experiments at 7 °C and 20 °C were carried out to validate the developed predictive model. In addition, to evaluate the model’s applicability to the duck meat, *Salmonella* cell counts were collected from duck meat at 15 °C and 25 °C. During storage, cell counts of *Salmonella* were collected using the procedures outlined above. *Salmonella* cell counts predicted by the developed model were then compared to the observed values. The root mean square error (*RMSE*) was used to evaluate observed and predicted data.

$$\mathrm{RMSE}=\sqrt{\sum {\left(predicted\;value-observed\;value\right)}^{2}/n}$$

where *n* represents the quantity of data points.

### 2.5. Reduction of Salmonella by Cooking

The representative cooking methods of chicken and duck meat were investigated through literature research [24,25]. The conditions of cooking time and temperature according to cooking method [the dry-heat (grilled or fried) or wet-heat (boiled, stewed, or steamed)] were investigated, and the appropriate or inappropriate cooking time was applied to the reduction experiment. Thus, their effects on the reduction of *Salmonella* cell counts were included as input variables in the simulation model. Since temperature and time are important in risk assessment, their conditions were set for complete cooking and incomplete cooking. Complete cooking, which means completely killed off *Salmonella* inoculated on chicken and duck meat, was supposed to be cooked for at least 1 min after reaching the internal temperature of 74 °C [26]. When the internal temperature was not reached at 74 °C, poultry meat products were undercooked, and that time was considered incomplete cooking.

### 2.6. Consumption Data for Chicken and Duck by Cooking Methods

The 2016 Korea National Health and Nutrition Examination Survey [27] provided information on the daily consumption ratio and the quantity of chicken and duck meat consumed. These data above were categorized with cooking methods (dry-heat and wet-heat) and calculated the ratio of consumption pattern by cooking methods. Using these data, the @Risk program determined the proper probability distribution for the daily consumption frequency and amounts by estimating cooking methods.

### 2.7. Distribution Temperature and Time

The temperatures and times for storing, displaying, and transporting chicken and duck meat were collected from personal communication with industry managers and the literature [28,29,30]. To obtain appropriate probabilistic distributions, the temperature and time data from each stage were evaluated with the @Risk program to obtain appropriate probabilistic distributions. These probabilistic distributions were used in the developed predictive models to simulate the fate of *Salmonella* during chicken and duck meat storage, display, and transport.

### 2.8. The Characterization of Risk Based on Dose-Response Model

Dose-response models were searched to estimate dose-responses after *Salmonella* ingestion. A Beta-Poisson model with *α* and *β* parameters of 0.89 and 4.4 × 10^5^, respectively, was used for this study [31].

$$P_{inf}=1-{\left(1+\frac{N}{\beta }\right)}^{-\alpha }$$

A simulation model was developed in Microsoft Excel (Microsoft Corporation, Seattle, WA, USA) using the initial *Salmonella* contamination level, predictive models, probabilistic distribution for temperature, time, consumption data, reduction of *Salmonella* after cooking, and a dose-response equation. The @Risk program ran the simulation model with the Monte Carlo method at 10,000 iterations. The most important factors affecting the incidence of *Salmonella* disease in the simulation model were assessed using @Risk.

### 2.9. Statistics

In SAS^®^ (Version 9.3, SAS Institute, Cary, NC, USA), *LPD* and *μ_max_* were examined using the GLM procedure. The mean kinetic parameters were compared using a pairwise *t*-test at α = 0.05.

## 3. Results

### 3.1. Salmonella Prevalence in Poultry during the Production Stage

**Figure 2:**
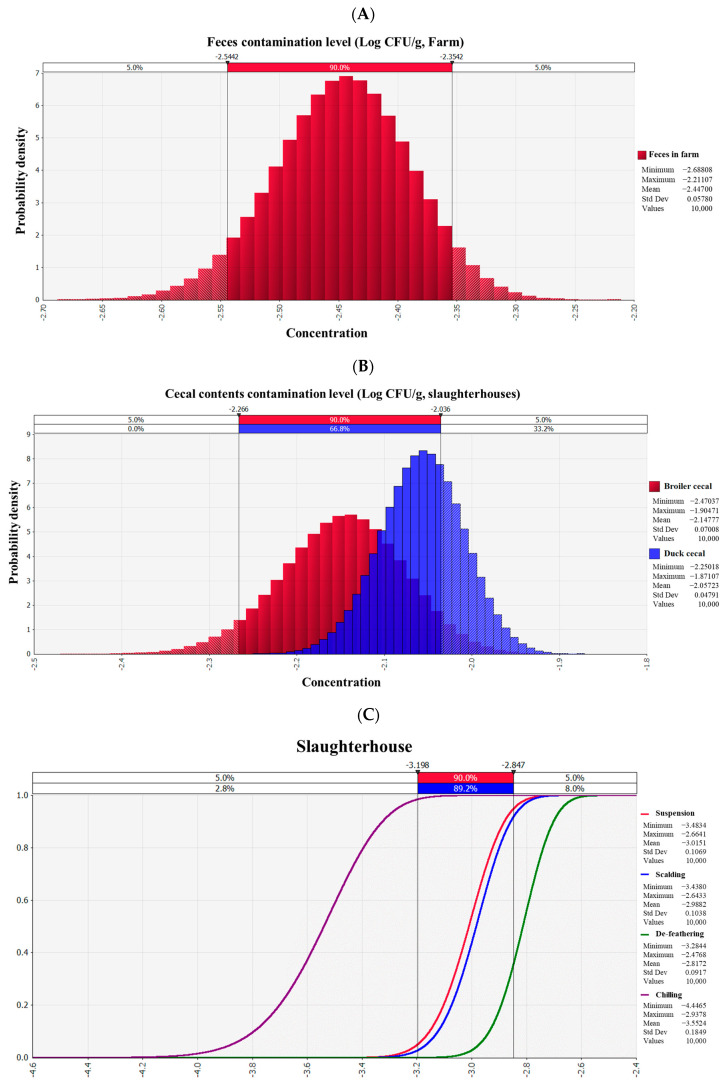

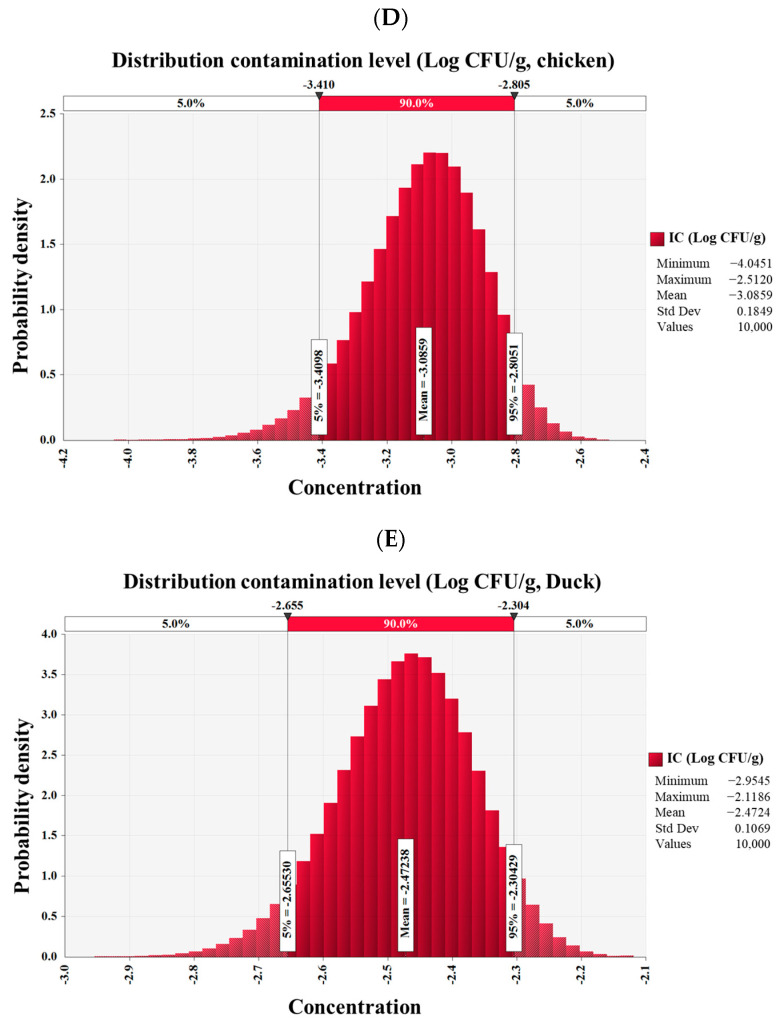
Probability of *Salmonella* contamination in farm-to-retail chicken and duck. (**A**) *Salmonella* contamination in farm feces. (**B**) *Salmonella* contamination in cecal contents of slaughterhouse. (**C**) Changes in *Salmonella* contamination levels by slaughterhouse stage. (**D**) *Salmonella* contamination levels in chicken at the distribution stage. (**E**) *Salmonella* contamination levels in duck at the distribution stage.

*Salmonella* was detected in 56 feces samples [8.5%; 95% confidence interval (CI) = 6.7–10.9%; 56/660] from farms, and *Salmonella* was not detected in the feed (n = 610) and eggs (n = 60) in the farms. As a result, the initial contamination level of the feces was estimated by describing the prevalence as Beta distribution [BetaRisk (57, 605)]. The probability distribution of the farm was then evaluated to determine the average contamination level of feces (−2.45 Log CFU/g; Figure 2A). In slaughterhouses, a total of 657 fecal samples were collected, including 237 chicken feces samples and 420 duck feces samples. Of the 420 duck fecal samples, 82 samples (19.5%; 95% CI = 16–23.6%) were contaminated with *Salmonella*, which was higher than that of chicken feces contamination level (16.0%; 95% CI = 11.9–21.2%; 38/237). Beta distribution with *Salmonella* cell counts of these data showed that average contamination levels in feces of chicken and duck were −2.2 Log CFU/g and −2.1 Log CFU/g, respectively (Figure 2B). In addition, the contamination level of *Salmonella* according to the producing step of chicken and duck slaughterhouses was estimated and analyzed. The average estimated contamination level of *Salmonella* slightly increased from −3.0 Log CFU/g to −2.9 Log CFU/g while passing through the scalding-defeathering step from the suspension step, and then it was lowered to −3.6 Log CFU/g after the chilling step (Figure 2C). 

### 3.2. Salmonella Prevalence in Poultry at the Distribution Stage

*Salmonella* was detected in 5 chicken samples (1.9%; 5/270) and 16 duck samples (7.8%; 16/205), and thus, the initial contamination level of chicken and duck meat were estimated using Beta distribution [BetaRisk (6, 266)] and [BetaRisk (17, 190)], respectively. In the equation presented by Sanaa et al. (2004) [32], the average initial *Salmonella* contamination level in chicken and duck meat was −3.1 Log CFU/g and −2.5 Log CFU/g, respectively (Figure 2D,E). This result showed that the contamination level of *Salmonella* in duck meat was higher than that in chicken meat, similar to the level of cecum contents in slaughterhouses. Since consumers purchase and consume chicken and duck meats at the distribution market, the risk assessment scenario in our study was designed from the distribution stage.

### 3.3. Predictive Models

**Figure 3:**
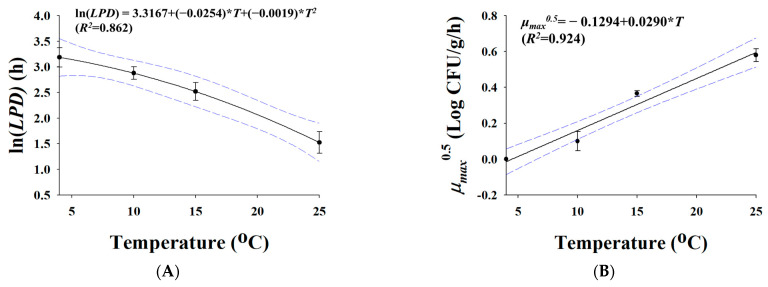
*Salmonella* lag phase (**A**) and growth rate (**B**) as a function of distribution temperature. Symbol, observed value; line, polynomial-fitted line. (**A**) ln(*LPD*), (**B**) $\sqrt{\mu_{max}\;}$.

Since chicken tenderloin had the highest growth of *Salmonella* levels among the chicken parts (whole chicken, legs, wings, and tenderloins), it was chosen as a representative poultry meat for the development of prediction models in this study. *Salmonella*-inoculated chicken tenderloin samples were stored at 4 °C, 10 °C, 15 °C, and 25 °C in order to develop predictive models. *Salmonella* cell counts steadily declined at 4 °C but increased at 10 °C, 15 °C, and 25 °C. Fitted kinetic parameters such as *LPD* and *μ_max_* were calculated from the *Salmonella* cell counts, and they are shown in Table 1. As temperature increased, *LPD*s went down (*p* < 0.05) from 25.03 to 4.79 h, while *μ_max_* values increased (*p* < 0.05) from −0.02 to 0.34 Log CFU/g/h. The *h*_0_ for 4 °C, 10 °C, 15 °C, and 25 °C, respectively, indicated a physiological state of −0.44, 0.20, 1.74, and 1.69. Figure 3 shows the secondary models for LN(*LPD*) and $\sqrt{\mu_{max}\;}$ as a function of temperature. With *R^2^* values ranging from 0.862 to 0.924, the created secondary models adequately describe the effect of temperature on the kinetic parameters (Figure 3). In validation, *RMSE* values were 0.352 and 0.340 at 7 °C and 20 °C, respectively. Because the *RMSE* is close to zero, the prediction of the model is correct. *RMSE* values for duck tenderloin were 0.346 and 0.271 at 15 °C and 25 °C, respectively. In addition, *RMSE* values for other parts of chicken were 0.384 (whole chicken), 0.377 (wings), and 0.447 (legs) at 15 °C. This result indicates that the model developed with chicken tenderloin can be used to describe *Salmonella* kinetic behavior in both chicken and duck.

### 3.4. Effects of Cooking Methods on Reducing Salmonella Cell Counts

**Figure 4:**
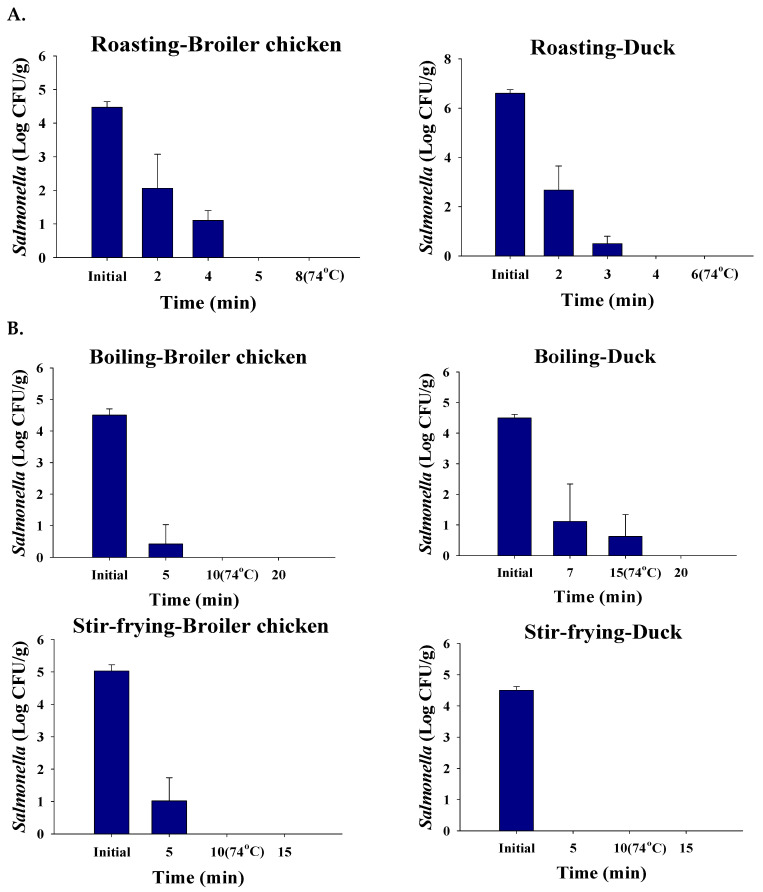
Methods of cooking shown to reduce *Salmonella* cell counts. (**A**) dry-heat cooking, (**B**) moist-heat cooking.

To investigate the reduction of *Salmonella* during the cooking process, the cooking methods were conducted with roasting (dry-heat cooking), boiling, and stir-frying (moist-heat cooking). When the *Salmonella*-contaminated (4.5 ± 0.2 Log CFU/g) poultry meat was roasted, *Salmonella* was not detected in the complete cook samples, which reached the internal temperature of 74 °C (Figure 4A), whereas when poultry meat was roasted in incomplete cooking, *Salmonella* remained 0.5–1.1 Log CFU/g after cooking for 3–4 min or below (Figure 4A). Thus, the incomplete cooking condition for roasting was determined to be less than 8 min for chicken and 6 min for duck. In the case of boiling, which is the most common moist-heat cooking method, *Salmonella* was detected (0.6 ± 0.7 Log CFU/g) in duck (whole) even though the internal temperature reached 74 °C (Figure 4B). These results indicate that the size of duck carcasses is larger than that of chicken. Thus, a longer time is required to completely kill off *Salmonella* inoculated on the poultry meat surface [24]. In the case of stir-frying, thin slices of chicken and duck meat were cooked so that the *Salmonella* inoculated poultry meat surface was not detected after 5 min (Figure 4C). Since *Salmonella* was detected until 5 min (1.0 ± 0.7 Log CFU/g in contaminated chicken meat), the complete cooking condition of chicken by moist-heat was determined to be 10 min. In addition, the incomplete cooking condition of duck by moist-heat was determined to be less than 20 min. The cooking methods and time affected the reduction of *Salmonella* cell counts. These results were used in the simulation model to calculate the reduction of *Salmonella* after cooking. Upon complete cooking conditions, *Salmonella* on the surface of poultry meat was assumed to be completely eliminated, whereas, in the case of the incomplete cooking conditions, 99% of *Salmonella* (2 Log CFU/g) on the surface of poultry meat was assumed as the control and applied in the simulation (Table 2).

### 3.5. Consumption and Cooking Method Ratios in Republic of Korea

According to the Korea National Health and Nutrition Examination Survey [27], the consumption amounts of respondents with chicken and duck were fitted with @Risk. The average chicken consumption amount was 115.7 g at 13.7% frequency, regardless of the cooking methods. In addition, the average chicken consumption amount was 154.5 g by lognormal distribution [RiskLognorm (158.26,203.31, RiskShift (−3.7516))] for dry-heat cooking at 36% of the ratio and 86.0 g by InvGauss distribution [RiskInvGauss (95.947,101.63, RiskShift (−9.9848))] for moist-heat cooking at 64% of the ratio. With the exception of smoked duck, the average duck consumption amount was 70.5 g at 2.84% frequency at the consumption stage. Smoked duck products were excluded from the consumption amount data because they are unpasteurized products which have to be heated before eating [21]. The appropriate probability distribution of the average amount of duck consumed by cooking methods was 81.3 g by lognormal distribution [RiskLognorm (88.864, 60.024, RiskShift (−7.5199))] for dry-heat cooking at 51% of the ratio and 57.9 g by exponential distribution [RiskExpon (58.692, RiskShift (−0.75665))] for moist-heat cooking at 49% of the ratio. 

### 3.6. Time and Temperature of Distribution

Poultry meat products, such as chicken and duck, are typically refrigerated during transport, storage, and market display. According to Park and Bahk (2017) [29], the minimum temperature of the refrigerated distribution vehicle was 2.12 °C and the maximum temperature was 12.54 °C. To obtain the probabilistic distribution, Uniform distribution (2.12, 12.54) was applied to the transport temperature from the processing plant to the market (Table 2). Since the poultry meat was transported within 30 min, 4 h, or 9 h of manufacture, the Pert distribution was chosen for analysis, with the following parameters: (0.5, 4, 9). After being transported to the market, poultry meat was stored between −2 °C and 5 °C (usually 2 °C) for 0–24 h. Therefore, the parameters (−2, 2, 5) were used to fit the Pert distribution of temperature, and the parameters (0, 24) were used to obtain the Uniform distribution of time. The market temperature and time for poultry meat ranged from −2 to 10 °C for 0–48 h (with a mean of 24 h), and thus, the probabilistic distribution was calculated using the Uniform distribution (−2, 10) for market temperature and the Pert distribution (0, 24, 48) for market display duration (Table 2). According to Jung (2011) [28], the market-to-home transport time and temperature ranged from 0.325 to 1.643 h at a mean of 18 °C (ranged from 10 to 25 °C). Pert distribution (10, 18, 25) and Uniform distribution (0.325, 1.643) were used to construct the probabilistic distribution (Table 2). Furthermore, the Uniform distribution (0, 72) was used to fit the data for home storage duration because the chicken was eaten within 72 h (the recommended storage duration). Lee et al. (2015) [30] proposed the Loglogistic distribution (−29.283, 33.227, 26.666, RiskTruncate (−5, 20)) to compare the temperature of poultry meat to the temperature of home refrigerators (Table 2).

### 3.7. Dose-Response Model

The Beta-Poisson model was chosen to estimate the dose responses in the host after intake of *Salmonella*. Teunis et al. (1999) [31] developed the parameters α = 0.89, β = 4.4 × 10^5^ (probability = 1 − [1 + D/β]^−α^).

### 3.8. Simulation and Risk Assessment

**Figure 5:**
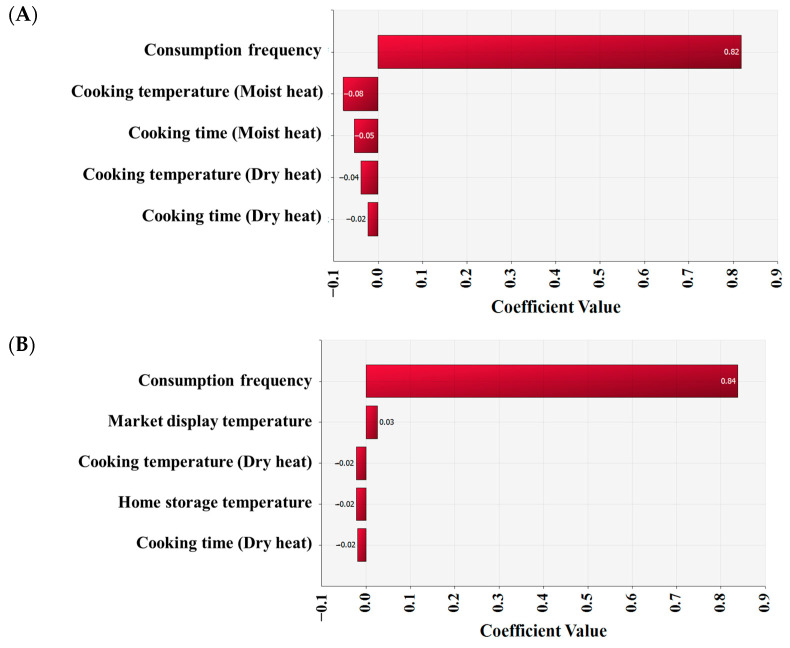
Coefficients of correlation for risk factors that influence the probability of *Salmonella* disease per person per day by poultry consumption. (**A**) Chicken, (**B**) Duck.

The simulation model involved home cooking from a store. As shown in Table 2, input models for the scenario included product (transport), market (storage and display), home (transport and storage), consumption (daily consumption frequency and average amounts), cooking method and reduction (dry- and moist-heat cooking), dose-response, and risk. The initial infection level of poultry meat was applied to develop predictive models that account for time and temperature to simulate *Salmonella* fates from market to home. After cooking, the final infection level (Log CFU/g) was applied to consumption. Final *Salmonella* survival counts were put to a dose-response model to calculate illness/person/day. As a result of the simulation, *Salmonella* contamination levels in poultry meat gradually decreased from the market to the consumer’s home. It indicates that the risk of *Salmonella* steadily decreases during distribution. At the initial contamination (IC) stage, the average *Salmonella* contamination level in chicken was estimated to be −3.1 Log CFU/g, and then gradually decreased until the market display stage (C2). Similarly, the average *Salmonella* contamination level in duck was calculated to be −3.2 Log CFU/g at the IC stage and significantly decreased to the C2 stage. The risk of *Salmonella* disease from home-cooked poultry meat was 3.0 × 10^−10^ (chicken) and 8.8 × 10^−11^ (duck) per person per day, indicating a low risk (Table 3). The simulation, which did not include cooking methods, revealed that the risk of *Salmonella* from chicken and duck consumption was 5.2 × 10^−8^ (1.7 × 10^2^-fold) and 1.4 × 10^−8^ (1.6 × 10^2^-fold), respectively (Table 3). When fitting without the effect of cooking methods, the risk of *Salmonella* foodborne illness was estimated to be higher. As shown in Figure 5, cooking and home storage may reduce the risk of *Salmonella* foodborne disease, whereas increasing consumption frequency may increase the risk.

## 4. Discussion

*Salmonella* causes 1.35 million illnesses, 420 fatalities, and 26,500 hospitalizations annually in the United States [34]. Furthermore, *Salmonella* infection is the third most common cause of food poisoning in South Korea, after EPEC and norovirus [4]. *Salmonella* is prevalent in a variety of foods, but poultry is a major source in Korea [20,35]. Nauta et al. (2005) [36] studied the impact of the manufacturing stage on risk management and emphasized chicken processing for food safety. Thus, it is critical to comprehend how to manage contamination from production to consumption, as well as how contamination can be reduced throughout slaughterhouse processing.

This study investigated potential contamination factors such as feed, feces, and environmental samples in slaughterhouses. Only 56 feces samples from the farms were contaminated with *Salmonella*. *Salmonella* was also found in the cecal contents of chicken (16%) and duck (19.5%) at slaughterhouses. These results indicate that *Salmonella* can be transmitted to the carcass surface through rinsing water and equipment surfaces. In other studies, feces contaminated with *Salmonella* is a significant source of environmental contamination and chicken infection [37], and these pathogens can be cross-contaminated through processing at slaughterhouses during the evisceration, defeathering, and rinsing steps [38,39]. In this study, *Salmonella* was found in feces at farms and slaughterhouses, which may cross-contaminate during processing, raising the level of contamination in the final food products. *Salmonella* prevalence increased from 4.3 to 21.5% during slaughterhouse evisceration, according to Xiao et al. (2021) [40]. During the evisceration stage, cross-contamination between carcasses occurred via tools and hands, leading to an increase in bacterial prevalence [41]. Chilling treatment, on the other hand, reduced *Salmonella* by 0.8 to 1.2 Log CFU/g, resulting in a 1.4-fold reduction in the final risk [40]. Using air-chilling, in particular, was the most effective processing strategy (34% reduction) and has the ability to reduce cross-contamination [42]. In our study, the contamination level of *Salmonella* increased throughout the suspension, scalding, and defeathering steps, but it was decreased after the chilling step in the slaughterhouse, which is in agreement with previous studies. Contaminated *Salmonella* can be controlled through the rinsing and chilling step in slaughterhouse. Therefore, it is necessary to prepare and strengthen the management plan for the rinsing water and the chilling step. The use of rinsing water containing a disinfectant and managing the storage temperature may be considered [43]. Especially, the chilling step stored at cool temperature is important to control *Salmonella* contaminating the poultry surface.

In this study, *Salmonella* contamination level in poultry meat at the stage of distribution was estimated to be higher than that at the stage of chilling. Although the poultry meat should be maintained and refrigerated at 10 °C or lower at all times during the transit to the consumer [21], the product can be exposed to inappropriate heat in the vehicle. Some products in a truck face remain at the proper temperature, while products in another section of the vehicle may be heated up during the transport. In general, the vehicle must be supplied with a good refrigerated system capable of keeping food at the proper temperature at all times during distribution [44]. To control *Salmonella*, poultry meats have to be stored at a low temperature throughout the production process before consumption. In another study, low temperatures were found to lower the risk of listeriosis in RTE products, and temperature at home was found to be an important factor in determining the risk of listeriosis in vacuum-packed products [45]. Therefore, a low temperature should be maintained to control *Salmonella* during transport and storage, both at retail and at home.

Although *Salmonella* is not particularly heat resistant, and most serotypes are killed by mild heat treatments for cooking, it can be detected in cooked poultry meat as a result of insufficient cooking. In the USA, 36 people were infected with *Salmonella* Enteritidis after eating undercooked frozen chicken products contaminated with *Salmonella* [46]. Except for the consumption frequency of poultry in our study (Figure 5), the sensitive analysis showed that cooking temperature was the most important factor in preventing salmonellosis. According to Xiao et al. (2021) [40], undercooking chicken meats increases the risk of infection by a factor of 20. In previous studies, cooking was also indicated as the most important factor in reducing the microbiological risk associated with poultry intake [20,46]. Dogan et al. (2019) [47] found that the rate of foodborne illness lowered to 0.12 cases per 100,000 person-years when chicken is cooked thoroughly but increased to 8.437 cases per 100,000 person-years when all chicken meats are undercooked. In our investigation, the average predicted probability of disease due to *Salmonella* ingestion from poultry meat cooked at home was calculated to be 3.0 × 10^−10^ (chicken) and 8.8 × 10^−11^ (duck) per person per day, which is a minimal risk. In S. Korea, most people consume poultry meat by cooking. Thus, the scenario of applying the cooking methods is thought to reflect reality and accuracy more. When the ratio of consumption patterns by cooking methods was changed, the risk of foodborne *Salmonella* illness was not different. Because the consumption of chicken (13.7%) and duck (2.84%) was so low, changes in consumption patterns due to cooking methods had no effect on the risk of *Salmonella* foodborne illness. Whereas, when the simulation considering only those who consumed poultry meat was estimated, the risk of *Salmonella* foodborne illness was affected by differences in consumption patterns. When the moist-heat cooking ratio was 100% (1.1 × 10^−8^), the risk of *Salmonella* foodborne illness from chicken meat was 3.3 times higher than when the dry-heat cooking ratio was 100% (3.3 × 10^−9^) (Table 3). Thus, changes in consumption patterns according to cooking methods affected the risk of foodborne *Salmonella* illness. 

## 5. Conclusions

*Salmonella* was mainly detected in feces from farms and slaughterhouses, and they remained on the surface of poultry meat due to cross-contamination during the production process. In farms and slaughterhouses, there is a need for methods to control the cross-contamination caused by feces. In the distribution stage from market to consumption after cooking, the risk for foodborne *Salmonella* illness through poultry consumption might be low. In the market, the prevalence of *Salmonella* in poultry meat was low, and most people in S. Korea consume poultry meat with cooking. The probability of *Salmonella* foodborne illness was low because the overall consumption frequency of chicken (13.7%) and duck (2.84%) was very low, regardless of consumption patterns or cooking methods. Despite increased concern about *Salmonella* foodborne disease from the consumption of poultry meat, the results of this study indicate that the risk of *Salmonella* disease from the consumption of cooked poultry meat is low in South Korea. Although this QMRA used the insufficient data evaluated under some assumptions, the risk of foodborne *Salmonella* illness can be re-estimated when additional data such as consumption data, temperature and time data, packaging methods, and reduction level by cooking methods are created.

## Figures and Tables

**Table 1:** Baranyi model kinetic parameters for *Salmonella* spp. in chicken and duck.

		Temperature (°C)			
		4	10	15	25
Kinetic parameters	*μ_max_*	−0.02 ± 0.01	0.01 ± 0.02	0.13 ± 0.02	0.34 ± 0.07
	LPD	25.03 ± 8.03	18.06 ± 3.93	12.79 ± 3.85	4.79 ± 1.53
*N*_0_		3.0 ± 0.1	3.4 ± 0.7	3.5 ± 0.8	3.5 ± 0.6
*R* ^2^		0.825 ± 0.084	0.551 ± 0.324	0.967 ± 0.026	0.959 ± 0.013

*μ_max_*: maximum specific growth rate (Log CFU/g/h), reflecting death and growth rates; LPD: lag phase duration (h), no cell count change in growth/death curve; *N*_0_: initial bacterial cell counts (Log CFU/g).

**Table 2:** @Risk simulation model and formulas used to estimate *Salmonella* spp. foodborne disease following intake of chicken and duck by cooking method.

Input Model	Unit	Variable	Formula	Reference
PRODUCT				
Pathogen contamination level				
*Salmonella* prevalence		PR	= RiskBeta(6, 266) for broiler chicken = RiskBeta(17, 190) for duck	This research; [33]
Level of initial contamination	CFU/g	C	= −LN(1 − PR)/25g	[32]
	Log CUF/g	IC	= LOG(C)	
TRANSPORTATION				
Time	h	Time_trans_	= RiskPert(0.5, 4, 9)	Personal communication ^a^
Temperature	°C	Temp_trans_	= RiskUniform(2.12, 12.54)	[23]
Growth		h_0_	= Average(LPD × growth rate), Fixed 0.7967	This research; [23]
	Log CFU/g	Y_0_	= Average(Y_0*i*_), Fixed 3.4	This research; [23]
	Log CFU/g	Y_end_	= Average(Y_end*i*_), Fixed 5.8	This research; [23]
		ln(q)	= LN(1/(EXP(h_0_) − 1))	This research; [23]
Growth rate	Log CFU/g/h	GR_trans_	= IF(Temp_trans_ > 4.46207, (0.0294 × (Temp_trans_ − 4.46207))^2^, 0)	This research; [23]
Level of *Salmonella*	Log CFU/g	C1	= IC + 1/(1 + EXP(−ln(q))) × (1 − (10 ^−|Y0−Yend|^/LN(10))) × GR_trans_ × Time_trans_	This research; [23]
MARKET				
Market storage				
Time	h	Time_mark-st_	= RiskUniform(0, 24)	Personal communication
Temperature	°C	Temp_mark-st_	= RiskPert(−2, 2, 5)	Personal communication
Growth		h_0_	= Average(LPD × growth rate), Fixed 0.7967	This research; [23]
	Log CFU/g	Y_0_	= Average(Y_0*i*_), Fixed 3.4	This research; [23]
	Log CFU/g	Y_end_	= Average(Y_end*i*_), Fixed 5.8	This research; [23]
		ln(q)	= LN(1/(EXP(h_0_) − 1))	This research; [23]
Growth rate	Log CFU/g/h	GR_mark-st_	= IF(Temp_mark-st_ > 4.46207, (0.0294 × (Temp_mark-st_ − 4.46207))^2^, 0)	This research; [23]
Level of *Salmonella*	Log CFU/g	C2	= C1 + 1/(1 + EXP(−ln(q)))×(1 − (10 ^−|Y0−Yend|^/LN(10))) × GR_mark-st_ × Time_mark-st_	This research; [23]
Market display				
Time	h	Time_mark-dis_	= RiskPert(0, 24, 48)	Personal communication
Temperature	°C	Temp_mark-dis_	= RiskUniform(−2, 10)	Personal communication
Growth		h_0_	= Average(LPD × growth rate), Fixed 0.7967	This research; [23]
	Log CFU/g	Y_0_	= Average(Y_0*i*_), Fixed 3.4	This research; [23]
	Log CFU/g	Y_end_	= Average(Y_end*i*_), Fixed 5.8	This research; [23]
		ln(q)	= LN(1/(EXP(h_0_) − 1))	This research; [23]
Growth rate	Log CFU/g/h	GR_mark-dis_	= IF(Temp_mark-dis_ > 4.46207, (0.0294 × (Temp_mark-dis_ − 4.46207))^2^, 0)	This research; [23]
Level of *Salmonella*	Log CFU/g	C3	= C2 + 1/(1 + EXP(−ln(q)))×(1 − (10 ^−|Y0−Yend|^/LN(10))) × GR_mark-dis_ × Time_mark-dis_	This research; [23]
TRANSPORTATION (vehicle)				
Time	h	Time_veh_	= RiskUniform(0.325, 1.643)	[28]
Temperature	°C	Temp_veh_	= RiskPert(10, 18, 25)	[28]
Growth		h_0_	= Average(LPD × growth rate), Fixed 0.7967	This research; [23]
	Log CFU/g	Y_0_	= Average(Y_0*i*_), Fixed 3.4	This research; [23]
	Log CFU/g	Y_end_	= Average(Y_end*i*_), Fixed 5.8	This research; [23]
		ln(q)	= LN(1/(EXP(h_0_) − 1))	This research; [23]
Growth rate	Log CFU/g/h	GR_veh_	= IF(Temp_veh_ > 4.46207, (0.0294 × (Temp_veh_ − 4.46207))^2^, 0)	This research; [23]
Level of *Salmonella*	Log CFU/g	C4	= C3 + 1/(1 + EXP(−ln(q)))×(1 − (10 ^−|Y0−Yend|^/LN(10))) × GR_veh_ × Time_veh_	This research; [23]
HOME				
Storage				
Time	h	Time_home_	= RiskUniform(0, 72)	Personal communication
Temperature	°C	Temp_home_	= RiskLogLogistic(−29.283, 33.227, 26.666, RiskTruncate(−5, 20))	[30]
Growth		h_0_	= Average(LPD × growth rate), Fixed 0.7967	This research; [23]
	Log CFU/g	Y_0_	= Average(Y_0*i*_), Fixed 3.4	This research; [23]
	Log CFU/g	Y_end_	= Average(Y_end*i*_), Fixed 5.8	This research; [23]
		ln(q)	= LN(1/(EXP(h_0_) − 1))	This research; [23]
Growth rate	Log CFU/g/h	GR_home_	= IF(Temp_home_ > 4.46207, (0.0294 × (Temp_home_ − 4.46207))^2^, 0)	This research; [23]
Level of *Salmonella*	Log CFU/g	C5	= C4 + 1/(1 + EXP(−ln(q)))×(1 − (10 ^−|Y0−Yend|^/LN(10))) × GR_home_ × Time_home_	This research; [23]
	CFU/g	C5_CFU/g_	= 10^C5^	
CONSUMPTION				
Daily consumption frequency for broiler chicken	%	ConFre	Fixed 13.7	[27] ^b^
		CF(0)	= 1 − 13.7/100	
		CF(1)	= 13.7/100	
		CF	= RiskDiscrete({0, 1}, {CF(0), CF(1)})	
Daily consumption frequency for duck	%	ConFre	Fixed 1.8	[27]
		CF(0)	= 1 − 1.8/100	
		CF(1)	= 1.8/100	
		CF	= RiskDiscrete({0, 1}, {CF(0), CF(1)})	
COOKING METHOD—Broiler chicken				
Dry heat cooking		Cook(dry)	= 36/100	[27]
Moist heat cooking		Cook(moist)	= 64/100	[27]
		Cook	= RiskDiscrete({1, 2}, {Cook(dry), Cook(moist)})	
Consumption at dry heat cooking	g	Consump_dry-cook_	= RiskLognorm(158.26, 203.31, RiskShift(−3.7516), RiskTruncate(0, 1256.4))	[27]
Consumption at moist heat cooking	g	Consump_moist-cook_	= RiskInvgauss(95.947, 101.63, RiskShift(−9.9848), RiskTruncate(0, 589.1))	[27]
	g	Consump	= IF(Cook = 1,Consump_dry-cook_, Consump_moist-cook_)	
Total consumption	g	Amount	= IF(CF = 0, 0, Consump)	
COOKING METHOD—Duck				
Dry heat cooking		Cook(dry)	= 51/100	[27]
Moist heat cooking		Cook(moist)	= 49/100	[27]
		Cook	= RiskDiscrete({1,2}, {Cook(dry), Cook(moist)})	
Consumption at dry heat cooking	g	Consump_dry-cook_	= RiskLognorm(88.864, 60.024, RiskShift(−7.5199), RiskTruncate(0, 294.3))	[27]
Consumption at moist heat cooking	g	Consump_moist-cook_	= RiskExpon(58.692, RiskShift(−0.75665), RiskTruncate(0, 461.7))	[27]
	g	Consump	= IF(Cook = 1, Consump_dry-cook_, Consump_moist-cook_)	
Total consumption	g	Amount	= IF(CF = 0, 0, Consump)	
REDUCTION—Broiler chicken				
Dry heat cooking		Reduce(dry)	= 36/100	[27]
Moist heat cooking		Reduce(moist)	= 64/100	[27]
		Reduce	= RiskDiscrete({1, 2}, {Reduce(dry), Reduce(moist)})	
Reduce(dry)—dry heat cooking				
Cooking time	h	Time_dry-cook_	= RiskPert(0.08, 0.13, 0.25)	This research
Food temperature during cooking	°C	Temp_dry-cook_	= RiskPert(74 × 0.8, 74, 74 × 1.2)	This research; [20]
	CFU/g	Reduce_dry-cook_	= IF(AND(Temp_dry-cook_ > 74, Time_dry-cook_ > 0.13), 0, C5_CFU/g_ × 0.01)	
Reduce(moist)—moist heat cooking				
Cooking time	h	Time_moist-cook_	= RiskPert(0.08, 0.17, 0.33)	This research
Food temperature during cooking	°C	Temp_moist-cook_	= RiskPert(74 × 0.8, 74, 74 × 1.2)	This research; [20]
	CFU/g	Reduce_moist-cook_	= IF(AND(Temp_moist-cook_ > 74, Time_moist-cook_ > 0.17), 0, C5_CFU/g_ × 0.01)	
Total reduction	CFU/g	Reduction	= IF({Reduce = 1, Reduce_dry-cook_, Reduce_moist-cook_)	
Final concentration	CFU/g	C6 (Cooked)	= IF(CF = 0, 0, Reduction)	This research
REDUCTION—Duck				
Dry heat cooking		Reduce(dry)	= 51/100	[27]
Moist heat cooking		Reduce(moist)	= 49/100	[27]
		Reduce	= RiskDiscrete({1, 2}, {Reduce(dry), Reduce(moist)})	
Reduce(dry)—dry heat cooking				
Cooking time	h	Time_dry-cook_	= RiskPert(0.03, 0.05, 0.1)	This research
Food temperature during cooking	°C	Temp_dry-cook_	= RiskPert(74 × 0.8, 74, 74 × 1.2)	This research; [20]
	CFU/g	Reduce_dry-cook_	= IF(AND(Temp_dry-cook_ > 74, Time_dry-cook_ > 0.05), 0, C5_CFU/g_ × 0.01)	
Reduce(moist)—moist heat cooking				
Cooking time	h	Time_moist-cook_	= Riskpert(0.12, 0.25, 0.33)	This research
Food temperature during cooking	°C	Temp_moist-cook_	= Riskpert(74 × 0.8, 74, 74 × 1.2)	This research; [20]
	CFU/g	Reduce_moist-cook_	= IF(AND(Temp_moist-cook_ > 74, Time_moist-cook_ > 0.25), 0, C5_CFU/g_ × 0.01)	
Total reduction	CFU/g	Reduction	= IF({Reduce = 1, Reduce_dry-cook_, Reduce_moist-cook_)	
Final concentration	CFU/g	C6 (Cooked)	= IF(CF = 0, 0, Reduction)	This research
DOSE-RESPONSE				
*Salmonella* amounts	CFU	D	= C6 × Amount	
Parameter of Beta-Poisson		α	Fixed, 0.89	[31]
		β	Fixed, 4.4 × 10^5^	[31]
RISK				
Probability of illness/person/day		Risk	= 1 − (1 + D/β)^−α^	

^a^ Personal communication with the merchandise manager. ^b^ Korea Disease Control and Prevention Agency.

**Table 3:** Probability of *Salmonella* spp. foodborne disease per person per day resulting from consumption of chicken and duck.

		50%	90%	95%	99%	Mean
Chicken	Probability of illness/person/day applied cooking methods	0	0	1.6 × 10^−9^	7.3 × 10^−9^	3.0 × 10^−10^
	Cooking methods not considered	0	8.3 × 10^−8^	2.7 × 10^−7^	10.0 × 10^−7^	5.2 × 10^−8^
Duck	Probability of illness/person/day applied cooking methods	0	0	0	1.5 × 10^−9^	8.8 × 10^−11^
	Cooking methods not considered	0	0	0	4.1 × 10^−7^	1.4 × 10^−8^

## Data Availability

Data is contained within the article.
